# Aberrant cerebral blood flow in tinnitus patients with migraine: a perfusion functional MRI study

**DOI:** 10.1186/s10194-021-01280-0

**Published:** 2021-06-29

**Authors:** Zhen-Gui Xu, Jin-Jing Xu, Yu-Chen Chen, Jinghua Hu, Yuanqing Wu, Yuan Xue

**Affiliations:** 1 Department of Otolaryngology, Nanjing Pukou Central Hospital, Pukou Branch Hospital of Jiangsu Province Hospital, No.166, Shanghe Road, 211899 Nanjing, China; 2 Department of Otolaryngology, Nanjing First Hospital, Nanjing Medical University, No.68, Changle Road, 210006 Nanjing, China; 3 Department of Radiology, Nanjing First Hospital, Nanjing Medical University, 210006 Nanjing, China

**Keywords:** migraine, chronic tinnitus, cerebral blood flow, functional MRI

## Abstract

**Purpose:**

Migraine is often accompanied with chronic tinnitus that will affect the cerebral blood flow (CBF) and exacerbate the tinnitus distress. However, the potential relationship between migraine and tinnitus remains unclear. This study will investigate whether aberrant CBF patterns exist in migraine patients with tinnitus and examine the influence of migraine on CBF alterations in chronic tinnitus.

**Materials and methods:**

Participants included chronic tinnitus patients (*n* = 45) and non-tinnitus controls (*n* = 50), matched for age, sex, education, and hearing thresholds. CBF images were collected and analyzed using arterial spin labeling (ASL) perfusion functional magnetic resonance imaging (fMRI). Regions with major CBF differences between tinnitus patients and non-tinnitus controls were first detected. The effects of migraine on tinnitus for CBF alterations were further examined. Correlation analyses illustrated the association between CBF values and tinnitus severity as well as between CBF and severity of migraine.

**Results:**

Compared with non-tinnitus controls, chronic tinnitus patients without migraine exhibited decreased CBF, primarily in right superior temporal gyrus (STG), bilateral middle frontal gyrus (MFG), and left superior frontal gyrus (SFG); decreased CBF in these regions was correlated with tinnitus distress. There was a significant effect of migraine on tinnitus for CBF in right STG and MFG. Moreover, the severity of migraine correlated negatively with CBF in tinnitus patients.

**Conclusions:**

Chronic tinnitus patients exhibited reduced CBF in the auditory and prefrontal cortex. Migraine may facilitate a CBF decrease in the setting of tinnitus, which may underlie the neuropathological mechanisms of chronic tinnitus comorbid with migraine.

## Introduction

Tinnitus is a common auditory disorder that affects approximately 10–15 % of adult populations, which severely impairs life quality of about 1–2 % of the general population [1, 2]. Chronic tinnitus patients suffer from secondary tinnitus symptoms or comorbidities, such as depression, anxiety, insomnia, and chronic pain [3, 4]. Furthermore, nearly 26–47 % of patients with tinnitus also suffer from migraine or headache[5]. The association between tinnitus and migraine has been described in prior studies. Therefore, migraine is a risk factor that may play an important role for tinnitus-related impairment in quality of life. It has been proposed that tinnitus and migraine overlap in their pathophysiological mechanism by sharing specific alterations in thalamocortical activity[6, 7]. However, the potential relationship between migraine and tinnitus still remains unclear.

Previous researches using functional magnetic resonance imaging (fMRI) have suggested that tinnitus is associated with aberrant brain functional changes in temporal cortex and non-auditory brain areas, including the prefrontal cortex, parahippocampus, insula and cerebellum[8]. Moreover, researches using cerebral perfusion, investigated via single-photon emission computed tomography (SPECT) and positron emission tomography (PET), showed that tinnitus patients exhibit decreased or increased cerebral blood flow (CBF) in widespread brain regions, such as temporal cortex, prefrontal cortex, and parahippocampal gyrus [9–11]. Arterial spin labeling (ASL) is used to evaluate CBF at resting state and could serve as a marker of functional activation albeit, which achieves a direct measure of regional CBF and independent of complicated calculations [12]. ASL perfusion fMRI, with higher resolution and accurate localization, has been applied to detect the CBF in various neurological or psychiatry disorders [13]. However, Emmert et al. did not observe any significant CBF changes between tinnitus patients and healthy controls using ASL [14], probably due to the limited sample size. Regarding migraine, previous studies have found that migraine patients exhibited hypoperfusion, hyperperfusion or no changes in whole-brain CBF [15–17]. Therefore, further research is required to investigate what regions reveal altered CBF in chronic tinnitus and to determine whether migraine is involved in the fluctuation of CBF values in these regions.

To address this issue, we raise the hypothesis that chronic tinnitus would exhibit aberrant CBF compared with non-tinnitus controls and that the migraine would have potential effect on the CBF in tinnitus patients. This study aimed to assess CBF differences between chronic tinnitus patients and matched controls using ASL perfusion fMRI and observed the effect of migraine on CBF changes in chronic tinnitus patients. We hypothesized that tinnitus patients would exhibit abnormal CBF in specific brain regions and migraine would exacerbate CBF abnormalities on tinnitus patients.

## Materials and methods

### Subjects and clinical data

 Study protocol was approved by the Research Ethics Committee of the Nanjing Medical University prior to study initiation. All the subjects provided written informed consent before any study procedures. Ninety-five subjects, including 45 patients with chronic bilateral tinnitus and 50 non-tinnitus controls (all right-handed and completed at least 8 years of education), were recruited through community health screening and newspaper advertisements and matched for sex, age, and education. According to the International Classification of Headache Disorders, Third Edition (beta version) (ICHD − 3 beta)[18], migraineurs without aura were diagnosed by a neurologist, then tinnitus patients were divided into two groups [20 with migraine (5 with chronic migraine and 15 with episodic migraine), and 25 without migraine]. Of the 20 patients with migraine headaches, 8 had headaches preferentially located at the right side of the head, 7 at the left side and 5 had bilateral headaches, or no side preference. The disease duration and attack frequency of migraine were also recorded. For the controls, they should have no personal or family history of migraine or other headaches. The Iowa version of the Tinnitus Handicap Questionnaires (THQ) [19] as well as a pure tone audiometry (PTA) examination was used to assess the tinnitus severity, tinnitus distress, and the hearing threshold. Any participants who had hearing loss (defined as thresholds ≥ 25 dB HL) at the frequencies of 0.25 kHz, 0.5 kHz, 1 kHz, 2 kHz, 4 kHz, and 8 kHz were excluded from the current study. Participants were excluded if they suffered from pulsatile tinnitus, hyperacusis or Meniere’s diseases, or if they had a past history of alcoholism, stroke, migraine, brain injury, anemia, Alzheimer’s disease, Parkinson’s disease, epilepsy, major depression or other neurological or psychiatric illness, MRI contraindications or severe visual loss, thyroid dysfunction, cancer, severe heart diseases and damaged liver/kidney function.

According to the Self-Rating Depression Scale (SDS) and the Self-Rating Anxiety Scale (SAS) (overall scores < 50, respectively), none of the participants had depression or anxiety. The pain intensity of migraine was measured by the visual analogue scale (VAS) and severity of migraine was measured by the Headache Impact Test-6 (HIT-6).

### MRI data acquisition

All participants were scanned using a 3.0 T MRI scanner (Ingenia, Philips Medical Systems, Netherlands). Foam padding and earplugs were used to reduce the head motion and scanner noise. The participants were instructed to rest quietly with their eyes closed and avoiding either falling asleep or making sudden head motions, and to not think of anything in particular during MRI scan. The subjects with migraine were not having pain during MRI scan. High resolution T1-weighted images were acquired using three-dimensional turbo fast echo (3D-TFE) sequence as follows: repetition time (TR) = 8.1 ms, echo time (TE) = 3.7 ms, thickness = 1 mm, slices = 170, gap = 0 mm, flip angle (FA) = 8°, field of view (FOV) = 256 × 256 mm^2^, and acquisition matrix = 256 × 256. The structural sequence took 5 min and 29 s. ASL images were obtained with a pseudo-continuous ASL (pcASL) sequence with a 2D fast spin-echo acquisition and background suppression using the parameters as follows: TR = 4000 ms, TE = 11 ms, slice thickness = 4 mm, label duration = 1650ms; post-label delay = 2000 ms, FA = 90°, FOV = 220 × 220 mm^2^, slices thickness = 4 mm, gap = 0.4 mm, matrix = 64 × 64. The ASL sequence took 4 min and 18 s.

### Imaging data processing

A voxel-based morphometry (VBM) approach was performed to estimate whole brain volumes using the VBM8 toolbox (http://dbm.neuro.uni-jena.de/vbm). DARTEL was used to improve inter-subject registration of the structural images. Briefly, cerebral tissues were segmented into gray matter (GM), white matter (WM), and cerebrospinal fluid by a unified segmentation algorithm [20]. Then, resulting GM and WM images were normalized to the MNI template, followed by smoothing using an 8-mm full width at half maximum (FWHM) Gaussian kernel. Finally, the resulting voxel-wise GM volume maps were entered as covariates in the ASL data analysis.

The ASL data were preprocessed to generate CBF maps using the ASL Perfusion MRI Signal Processing Toolbox (ASLtbx), which is based on SPM12 (http://www.fil.ion.ucl.ac.uk/spm/) [21]. All images were first rearranged and adjusted to correct head movement. Next, a nonlinear transformation was performed on the CBF images of healthy controls, which were co-registered with the PET- perfusion template in Montreal Neurological Institute (MNI) space. The MNI-standard CBF template was defined as the average co-registered CBF images of healthy controls. The CBF images of all participants were then co-registered to the MNI-standard CBF template. Every co-registered CBF was removed from the non-brain tissue. Then a spatial smoothing with an isotropic Gaussian at FWHM of 6 mm^3^ was followed. Finally, normalization was performed by dividing the CBF per voxel by the average CBF across the entire brain [22]. The relative CBF (rCBF) was used in the whole analysis. None of the participants was excluded from the study due to head movement exceeding 2.0 mm of maximum translation in any of the x, y, and z directions or 2.0°of the maximum rotation around the three axes.

### Statistical analyses

Clinical measures were analyzed using Statistical Package for Social Sciences (SPSS) statistics software package version 20.0 (IBM Corp., Armonk, NY, USA). The statistical significance level was set at *p* < 0.05, two-tailed. One-way analysis of variance (ANOVA) was used to calculate the difference among the three groups followed by a post hoc test (t-test for means and χ2-test for proportions) between tinnitus patients with migraine and without migraine. For some non-normal distributed parameters, such as PTA, we used Mann-Whitney U test for between-group analysis.

A one-way analysis of variance (ANOVA) was then performed to determine between-group differences in brain volumes, with age, gender, and education as the nuisance covariates. Between-group rCBF differences were also calculated via one-way ANOVA in SPM12 with age, gender, education level and GM volume as the nuisance covariates. Significant thresholds were corrected using false discovery rate (FDR) criterion and set at *p* < 0.01. A full-factorial model was utilized to detect potential interaction effects of tinnitus and migraine on rCBF differences. Full factorial analysis was conducted to analyse the main effects and interaction effect of tinnitus and migraine using SPM12 software. Specifically, the between-subject factors included tinnitus group and migraine group. Significant thresholds were corrected using cluster-level family-wise error (FWE), and the threshold was set at *p* < 0.01.

The relationships between aberrant rCBF and each clinical characteristic were further investigated. Firstly, regions showing significant differences between groups were extracted. Then the mean z-values of aberrant rCBF region mask were calculated within every subject. Pearson correlation analysis between the mean z-values and each clinical characteristic were performed using SPSS software. Partial correlations were calculated with age, sex, education, GM volume, and average hearing thresholds as the nuisance covariates. *P* < 0.05 was considered statistically significant.

## Results

### Demographics and clinical data

**Fig. 1:**
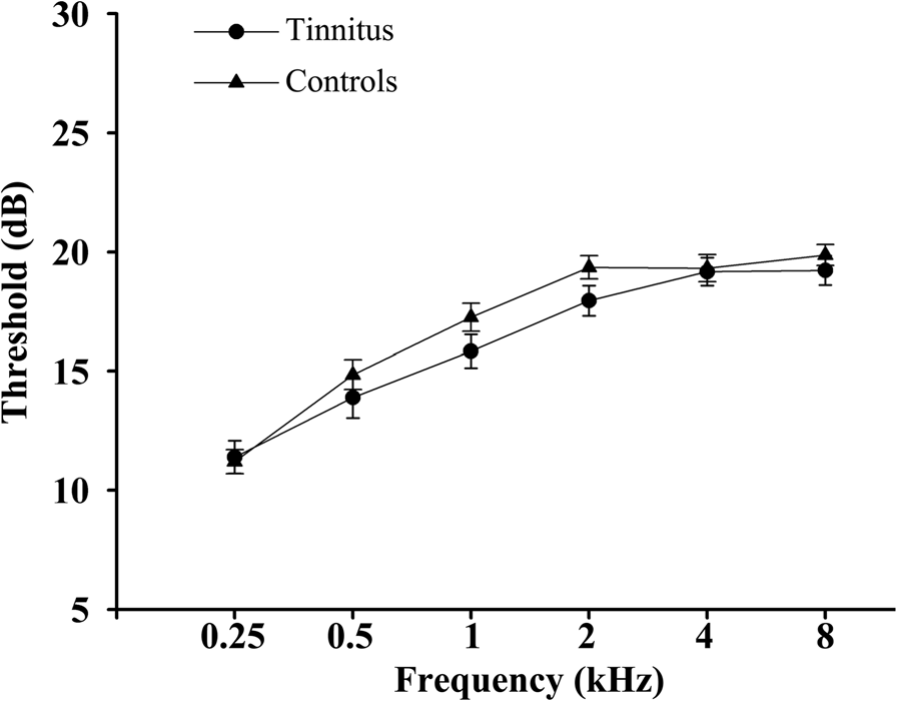
Mean hearing thresholds of the chronic tinnitus patients and non-tinnitus controls. Data are presented as Mean ± SEM

Demographics and clinical characteristic data of all the chronic tinnitus patients and non-tinnitus controls were summarized in Table 1. No significant differences were found in terms of age, gender, education level, and hearing thresholds. Moreover, there were no significant differences in each frequency of auditory thresholds between two groups (Fig. 1). Furthermore, no significant differences were observed in terms of age, gender, education level, and hearing thresholds between tinnitus patients with and without migraine (Table 2). However, tinnitus patients with migraine had worse THQ scores than patients without migraine (*p* < 0.05).

**Table 1:** Demographic and clinical characteristics of tinnitus patients and non-tinnitus controls

	Tinnitus patients (*n* = 45)	Non-tinnitus controls (*n* = 50)	*p* value
Age (years)	49.82 ± 11.51	47.36 ± 12.39	0.320
Gender (male: female) Education levels (years) Tinnitus duration (months) THQ score HIT-6 score VAS score Hearing thresholds (left) Hearing thresholds (right) Hearing thresholds (average)	15:30 12.56 ± 2.95 39.20 ± 37.94 52.01 ± 13.77 62.00 ± 2.36 5.50 ± 1.43 16.06 ± 2.65 16.52 ± 3.03 16.24 ± 2.52	20:30 12.84 ± 3.13 -- -- -- -- 16.95 ± 2.49 17.00 ± 2.34 16.92 ± 1.62	0.501 0.651 -- -- -- -- 0.094 0.385 0.126

Data are represented as Mean ± SD. The PTA from both ears was averaged. PTA, puretone audiometry; THQ, Tinnitus Handicap Questionnaires; HIT-6, migraine impact test-6; VAS, visual analogue scale

**Table 2:** Demographic and clinical characteristics of tinnitus patients with and without migraine

	Tinnitus with migraine (*n* = 20)	Tinnitus without migraine (*n* = 25)	*p* value
Age (years)	49.00 ± 12.26	50.48 ± 11.08	0.673
Gender (male: female) Education levels (years) Tinnitus duration (months) THQ score HIT-6 score VAS score Migraine duration (months) Frequency (d/m) Hearing thresholds (left) Hearing thresholds (right) Hearing thresholds (average)	5:15 12.60 ± 3.10 49.20 ± 41.14 56.78 ± 12.98 62.00 ± 2.36 5.50 ± 1.43 42.40 ± 35.92 8.75 ± 2.92 16.59 ± 2.80 16.54 ± 2.71 16.17 ± 2.04	10:15 12.52 ± 2.89 31.20 ± 33.90 48.20 ± 13.42 -- -- -- -- 15.63 ± 2.51 16.50 ± 3.32 16.29 ± 2.88	0.289 0.929 0.115 0.036^*^ -- -- -- -- 0.230 0.964 0.869

Data are represented as Mean ± SD. The PTA from both ears was averaged. PTA, puretone audiometry; THQ, Tinnitus Handicap Questionnaires; HIT-6, migraine impact test-6; VAS, visual analogue scale. ^*^p < 0.05

### Structural data

There were no significant differences in the comparisons of the whole-brain volumes (GM volume, WM volume and brain parenchyma volume) between chronic tinnitus patients and controls (Table 3). Moreover, we also observed no significant differences of whole-brain volumes between tinnitus patients with migraine and without migraine. After Monte Carlo simulation correction, no suprathreshold voxel-wise difference in the GM and WM volumes between chronic tinnitus patients and controls was observed.

**Table 3:** Comparisons of volumes between tinnitus patients and non-tinnitus controls

Brain volume	Tinnitus patients	Non-tinnitus controls	*p* value
Gray matter volume (% of TIV)	31.78 ± 2.01	32.39 ± 2.01	0.143
White matter volume (% of TIV)	29.59 ± 1.54	29.53 ± 1.62	0.861
Brain parenchyma volume (% of TIV)	61.37 ± 2.95	61.92 ± 3.16	0.382

Data are presented as mean ± SD. TIV, total intracranial volume.

### Group rCBF differences

**Fig. 2:**
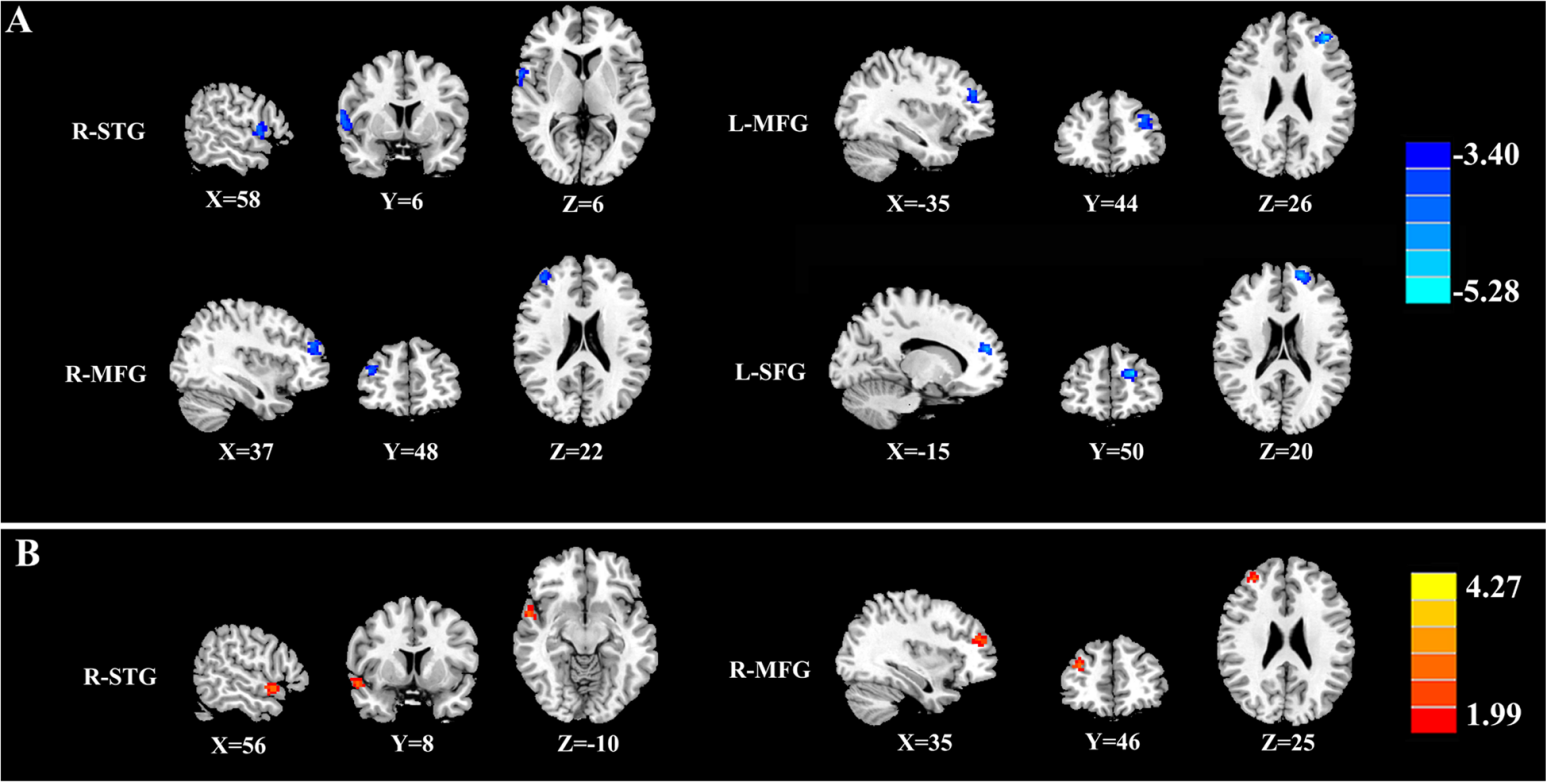
The rCBF differences between the chronic tinnitus patients and non-tinnitus controls. (**A**) The tinnitus patients exhibited decreased rCBF in the right superior temporal gyrus (STG), left and right middle frontal gyrus (MFG), and left superior frontal gyrus (SFG) (*P* < 0.01, FWE corrected); (**B**) The interaction effect of migraine and tinnitus in the right STG and right MFG (*P* < 0.01, FWE corrected)

**Fig. 3:**
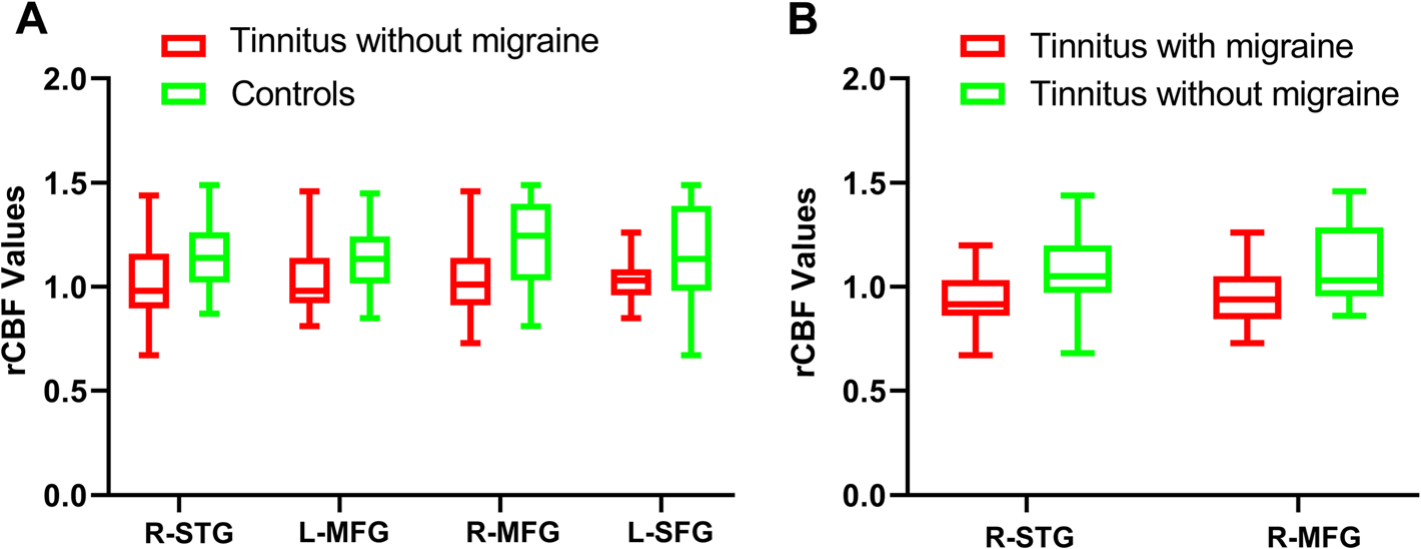
The rCBF values for different groups. (**A**) The rCBF values of the chronic tinnitus patients without migraine and non-tinnitus controls in the right STG, left MFG, right MFG, and left SFG (*P* < 0.01). (**B**) The interaction effect of migraine on tinnitus in the right STG and right MFG (*P* < 0.01)

The rCBF differences between the chronic tinnitus patients without migraine and non-tinnitus controls were shown in Fig. 2 A and Table 4. The tinnitus patients exhibited decreased rCBF, primarily in the right superior temporal gyrus (STG), bilateral middle frontal gyrus (MFG), and left superior frontal gyrus (SFG) (*P* < 0.01, FWE corrected). These reductions are due solely to the presence of tinnitus and not to the presence of subjects with migraine and not in the controls. The interaction effect of migraine on tinnitus was significant in the right STG and right MFG (Fig. 2B; Table 5) (*P* < 0.01, FWE corrected). When the tinnitus patients had migraine, the rCBF would be decreased, especially in the right STG and right MFG. The rCBF values for different groups were shown in Fig. 3.

**Table 4:** Brain regions with significant differences in CBF between tinnitus without migraine and non-tinnitus controls

Brain regions	BA	Peak MNI coordinates x, y, z (mm)	Peak T value	Cluster size (Voxels)
R superior temporal gyrus L middle frontal gyrus	22 10	58, 6, 6 -35, 44, 26	-4.3271 -4.8734	159 169
R middle frontal gyrus	10	37, 48, 22	-4.2989	143
L superior frontal gyrus	10	-15, 50, 20	-4.5877	215

Thresholds were set at a corrected *p* < 0.01 corrected by FWE criterion

**Table 5:** Regions showing interaction effects of tinnitus and migraine on CBF

Brain regions	BA	Peak MNI coordinates x, y, z (mm)	Peak T value	Cluster size (Voxels)
R superior temporal gyrus R middle frontal gyrus	22 10	56, 8, -10 35, 46, 25	3.8773 3.9324	109 95

Thresholds were set at a corrected *p* < 0.01 corrected by FWE criterion

### Correlation analysis

**Fig. 4:**
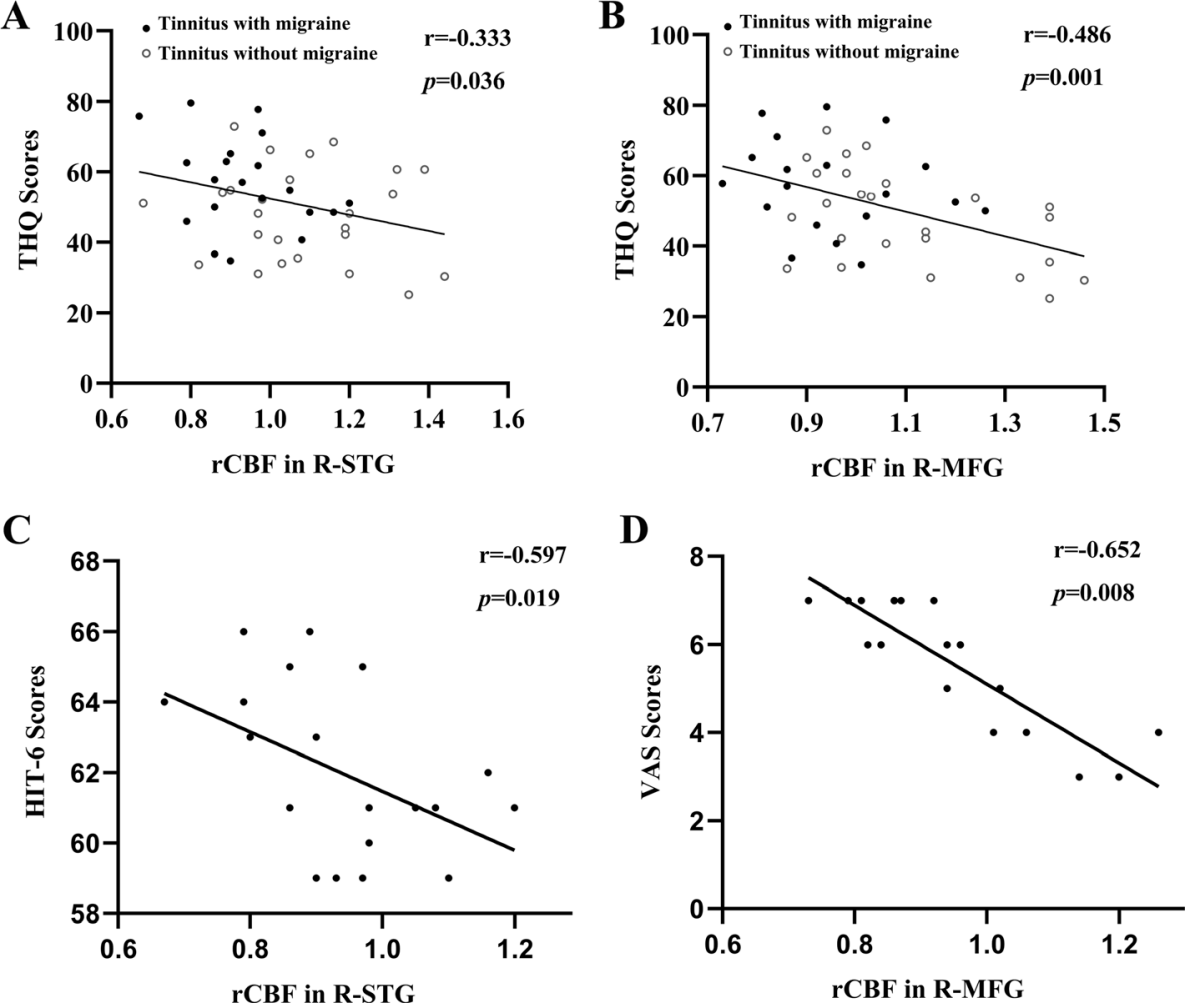
The significant correlations between the rCBF changes and the clinical data. (**A**) rCBF in the right STG was negatively associated with THQ scores (r=-0.333, *p* = 0.036) in tinnitus patients with and without migraine; (**B**) rCBF in the right MFG was negatively associated with THQ scores (r=-0.486, *p* = 0.001) in tinnitus patients with and without migraine; (**C**) rCBF in the right STG was inversely correlated with the HIT-6 scores (r=-0.597, *p* = 0.019) in tinnitus patients with migraine; (**D**) rCBF in the right MFG was inversely correlated with the VAS scores (r=-0.652, *p* = 0.008) in tinnitus patients with migraine

The significant correlations between the rCBF changes and the clinical data were depicted in Fig. 4. Regarding the tinnitus characteristics, the rCBF in the right STG and the right MFG was negatively associated with THQ scores, respectively (r=-0.333, *p* = 0.036; r=-0.486, *p* = 0.001). Regarding the correlations between migraine and rCBF values, we observed that the HIT-6 scores were inversely correlated with the rCBF in the right STG (r=-0.597, *p* = 0.019). Moreover, the VAS scores were inversely correlated with the rCBF in the right MFG (r=-0.652, *p* = 0.008).

## Discussion

The current study explored for the first time the associations between migraine and tinnitus using ASL perfusion fMRI. Compared to non-tinnitus controls, tinnitus patients exhibited reduced CBF in temporal and prefrontal cortex, which was associated with tinnitus distress. Interestingly, we also observed that migraine exacerbated rCBF reduction in tinnitus patients, and the severity of migraine was linked with decreased rCBF.

Chronic tinnitus has been associated with GM changes in widespread brain regions [23]. We expected to observe brain atrophy in these patients, however, neither regional nor whole-brain atrophy was observed in our tinnitus patients compared to matched controls. It is possible that the absence of any hearing loss in our tinnitus population may be one reason for the different results. In addition, the MR technique and analytical method may be less sensitive for detecting subtle structural changes.

In this study, main effects of chronic tinnitus were primarily in auditory cortex and prefrontal cortex. Our tinnitus patient group exhibited decreased rCBF primarily in the right STG, which was associated with THQ scores. Dysfunction of the temporal gyrus is associated with affective disturbance, which has been proved to be linked with migraine [24–26]. The tinnitus comorbid with migraine may lead to more complex dysfunction in the cortico-limbic network. Moreover, prior fMRI researches have revealed the associations between aberrant neuronal activity and functional connectivity of the STG and tinnitus distress[27, 28]. However, Emmert et al. found no significant rCBF alterations within the auditory cortex in tinnitus patients using ASL [14]. Moreover, previous studies using SPECT or PET did not detect reduced cerebral perfusion in primary auditory cortex [11, 10, 9], which was different from our current results. We infer that different sample sizes and imaging modalities may contribute to the discrepancy. Furthermore, the prefrontal cortex, including the MFG and SFG, exhibited decreased rCBF in tinnitus patients compared with non-tinnitus controls. The prefrontal cortex plays a critical role in emotional processing and executive function [29]. Disrupted brain activity was observed within the executive control of attention network, including the MFG and SFG[8]. Previous resting-state fMRI studies have also pointed out the abnormalities of the prefrontal cortex could act as a direct mechanism of tinnitus chronification [30, 31]. Therefore, these findings suggested that the rCBF alterations in the prefrontal cortex may be one important brain characteristic of chronic tinnitus. Nevertheless, the specific clinical implication for reduced rCBF in the MFG and SFG is unclear and requires to be further investigated.

Previous studies showed that patients with migraine exhibited reduced perfusion in temporal and prefrontal cortex [15, 16]. Our data support and extend these findings by identifying a significant effect of migraine on tinnitus for rCBF in the STG and MFG, indicating that the existence of migraine accelerates the decrease in rCBF in the setting of tinnitus, resulting in impaired attention and executive function. In addition, the severity of migraine is negatively associated with decreased rCBF in tinnitus patients, which was consistent with some researches about migraine without aura attacks [15–17]. We speculate that increased severity of migraine will aggravate the tinnitus distress and severity, reflecting the aberrant perfusion in specific brain regions. Nevertheless, the association of the migraine with the chronic tinnitus has not been substantially elucidated and also needs to be corroborated in future studies.

Several limitations should be acknowledged in the current study. Firstly, due to the strict inclusion and exclusion criteria, our sample size was not large enough that may affect the statistical reliability of our results. We will further expand the sample size and obtain more reliable results in future studies. Secondly, the characteristics of migraine were just assessed using HIT-6 and VAS scores in this study. More neuropsychological tests are needed to assess the symptom of the migraine, such as the Migraine Disability Assessment Questionnaire (MIDAS) [32] and Visual Light Sensitivity Questionnaire-8 (VLSQ-8) [33]. Furthermore, although we try to diminish the MR scanner noise using earplugs, the tinnitus patients cannot be completely prevented from scanner noise that probably affects the rCBF perfusion to varying degree. This confounding factor should be taken into account for all perfusion fMRI studies related to the auditory system. Finally, we only measured the rCBF changes within each brain regions but did not prove the possibility of rCBF functional connectivity among different brain areas. A data-driven approach to whole-brain rCBF connectivity analysis will be considered in our future study.

In conclusion, this study provided evidence that chronic tinnitus patients exhibited reduced rCBF in auditory and prefrontal cortex, and that decreased rCBF in tinnitus patients was associated with tinnitus severity. Migraine may exacerbate rCBF reduction in tinnitus patients, and the severity of migraine was negatively associated with rCBF levels. These findings suggest that abnormalities in rCBF perfusion may contribute to the pathophysiological mechanisms of chronic tinnitus with migraine condition. The co-occurrence of tinnitus and migraine is not purely coincidental but both disorders may be linked by shared neuropathological mechanisms.

## Data Availability

Clinical, neuroimaging and statistical data will be available upon request from any qualified investigator.

## Abbreviations

fMRI: functional magnetic resonance imaging
SPECT: single-photon emission computed tomography
PET: positron emission tomography
CBF: cerebral blood flow
ASL: arterial spin labeling
GM: gray matter
WM: white matter
THQ: Tinnitus Handicap Questionnaires
PTA: pure tone audiometry
SDS: Self-Rating Depression Scale
SAS: Self-Rating Anxiety Scale
VAS: visual analogue scale
HIT-6: Headache Impact Test-6
FDR: false discovery rate
FWE: family-wise error
STG: superior temporal gyrus
MFG: middle frontal gyrus
SFG: superior frontal gyrus
